# Protective effects of polyunsatutared fatty acids supplementation against testicular damage induced by intermittent hypobaric hypoxia in rats

**DOI:** 10.1186/s12929-015-0112-8

**Published:** 2015-01-23

**Authors:** Rodrigo L Castillo, Andrea B Zepeda, Stefania E Short, Elías Figueroa, Eduardo Bustos-Obregon, Jorge G Farías

**Affiliations:** Pathophysiology Program, Institute of Biomedical Sciences, Faculty of Medicine, University of Chile, Santiago, Chile; Departamento de Ingeniería Química, Facultad de Ingeniería y Ciencias, Universidad de La Frontera, Casilla 54-D, Temuco, Chile; Department of Biochemical and Pharmaceutical Technology, School of Pharmaceutical Sciences, University of São Paulo, Av. Prof. Lineu Prestes, 580 – Bloco 16, São Paulo, SP 05508-900 Brazil; School of Aquaculture, Catholic University of Temuco, Faculty of Natural Resources, Temuco, Chile; Anatomy and Developmental Biology Program, Faculty of Medicine, University of Chile, Santiago, Chile

**Keywords:** Intermittent hypoxia, Testis, Oxidative stress, PUFA, SIRTUIN 1

## Abstract

**Background:**

Intermittent hypobaric hypoxia (IHH) induces changes in the redox status and structure in rat testis. These effects may be present in people at high altitudes, such as athletes and miners. Polyunsaturated fatty acids (PUFA) can be effective in counteracting these oxidative modifications due to their antioxidants properties. The aim of the work was to test whether PUFA supplementation attenuates oxidative damage in testis by reinforcing the antioxidant defense system. The animals were divided into four groups (7 rats per group): normobaric normoxia (~750 tor; pO2 156 mmHg; Nx); Nx + PUFA, supplemented with PUFA (DHA: EPA = 3:1; 0.3 g kg^−1^ of body weight per day); hypoxic hypoxia (~428 tor; pO2 90 mmHg; Hx) and, Hx + PUFA. The hypoxic groups were exposed in 4 cycles to 96 h of HH followed by 96 h of normobaric normoxia for 32 days. Total antioxidant capacity (FRAP) and lipid peroxidation (malondialdehyde, MDA) in plasma and reduced (GSH)/oxidized glutathione (GSSG) ratio, tissue lipid peroxidation (TBARS) and antioxidant enzymes activity were assessed at the end of the study in testis. Also, SIRTUIN 1 and HIF-1 protein expression in testis were determined.

**Results:**

IHH increased lipid peroxidation in plasma and HIF-1 protein levels in testis. In addition, IHH reduced FRAP levels in plasma, antioxidant enzymes activities and SIRTUIN 1 protein levels in testis. PUFA supplementation attenuated these effects, inducing the increases in FRAP, in the antioxidant enzymes activity and HIF-1 levels.

**Conclusions:**

These results suggest that the IHH model induces a prooxidant status in plasma and testis. The molecular protective effect of PUFA may involve the induction of an antioxidant mechanism.

## Background

Humans at high altitudes are exposed to hypobaric hypoxia, a condition where reduced barometric pressure decreases oxygen availability [1] resulting in lower circulating oxygen levels [2]. Populations living at high altitudes are exposed to chronic hypobaric hypoxia whereas activities such as mountain sports, tourism and certain-jobs (i.e. customs agents, mining, educational and health workers) performed in areas over 1,500 meters above sea level (asl) involve intermittent exposure to hypobaric hypoxia (IHH) [3-5].

At high altitudes, oxidative stress is higher than at sea level under normobaric conditions, due to a combination of factors such as temperature fluctuation, increased UV light intensity and metabolic rate, low blood oxygenation and localized anoxia/reoxygenation [6]. Due to their ubiquitous nature, increased reactive oxygen species (ROS) may cause cell membrane damage through lipid peroxidation, protein oxidation, DNA damage, and consecutively impaired ATP production [7,8]. Oxidative stress may also produce cell swelling, decrease cell membrane fluidity, prevent the maintenance of ionic gradients and lead to tissue inflammation, which can result in structural and functional detrimental effects [6]. Chronic and IHH have been associated with an increase of oxidative damage in testis [9]. IHH induces higher levels of lipid peroxidation, reduces glutathione reductase activity, and decreases the epididymal sperm count [10]. These changes have been partially attenuated by the supplementation of antioxidants such as melatonin and ascorbate [10,11].

Studies and clinical intervention trials have demonstrated a probable protective role of dietary fish oils and n-3 polyunsaturated fatty acids (PUFA) in cardiovascular diseases [12-14]. The beneficial effects have been attributed to the improvement of vascular endothelial cell function, enhancement of vascular reactivity and compliance, modulation of lipid metabolism and reduction of inflammatory cytokine production [15]. Docosahexaenoic acid (DHA, C22:6n-3), a major n-3 PUFA, is generally acknowledged as a physiologically active component of these effects [16]. The cellular and molecular mechanisms underlying the beneficial effects of DHA on various cell types during ischemia-reperfusion (IR) injury, especially cardiomyocytes, vascular endothelial, and smooth muscle cells, have been extensively studied [17,18]. These protective mechanisms include the induction anti-inflammatory transcriptional pathways, the decrease in intracellular Ca^2+^levels, the suppression of vascular proliferation, and the improvement of cell membrane integrity [19,20]. Indeed, PUFA may induce an antioxidant mechanism through the induction of nuclear factor erythroid 2-related factor 2 (Nrf2), a master transcriptional factor for antioxidant genes and glutathione synthesis in vascular tissue [21]. In testis, PUFA may be beneficial for improving sperm motility and viability following acute doxorubicin-induced injury [22]. However, the effects of PUFA on oxidative stress markers and antioxidant mechanisms have not been well characterized in testis.

Therefore, in an established rat model of IHH we hypothesized that PUFA supplementation attenuates oxidative damage in testis by reinforcing the antioxidant defense system.

## Methods

### Experimental design

All animal care and procedures complied with the principles of animal care outlined by the National Society Laboratory and the Medical Research, and the Guide for the Care and Use of Laboratory Animals (Institute of Animal Laboratory Resources, 1996), and were approved by the Faculty of Medicine’s Bioethics Committee. (Study number: CBA# 0627 FMUCH). All experiments were performed in adult male Wistar rats (n = 28). The animals were randomly divided into four equal groups: normobaric normoxia (~750 tor; pO2 156 mmHg; Nx, n = 7); Nx + PUFA (n = 7), supplemented with PUFA (DHA: EPA = 3:1; 0.3 gkg^−1^d^−1^) via gavage; hypoxic hypoxia (~428 tor; pO2 90 mmHg; Hx, n = 7) and, Hx + PUFA (n = 7). The hypoxic groups were exposed in 4 cycles to 96 h of hypobaric hypoxia followed by 96 h of normobaric normoxia for 32 days. The desired pressure inside the hypobaric chamber was achieved by pressure changes simulating altitude increases of 150 m per minute. The animals in the Nx groups were lodged in the same room as the Hx (22°C). The 4 groups received the same amount of daily food and drinking water (15 g of standard pellet meals; water ad libitum). Body weight was determined at the beginning of the study and then every other day, and at the end of each period. The PUFA supplementation was maintained for the 32 days of the study. At the end of the study and after blood samples and tissue extraction, the rats were euthanized by cervical dislocation in a room separated from the remaining animals.

### Blood sample preparation

Animals from both groups were injected intraperitoneally with 0.2 ml of ketamine to numb them; subsequently the cardiac puncture was performed using the Burhoe’s Method modified, in accordance with the handbook “The Rat in Laboratory Investigation”. One ml of blood was collected from each and centrifuged (Centrifuge 5804R, Eppendorf AG, No. 5805XK634757) at 489 × *g* for 35 min to separate plasma from blood cells. The plasma was separated in 100 μl aliquots and kept at −80°C until use.

### Determination of antioxidant capacity and lipid peroxidation in plasma

Plasma antioxidant capacity was assessed by measuring the ability of plasma compounds to reduce ferric (Fe^+3^) to ferrous iron (Fe^+2^) (Ferric Reducing Ability of Plasma assay, FRAP). The Fe^+2^-III/TPTZ-complex was detected at light absorbance 594 mm (spectrophotometer method), with a detection limit of 10 μmol/l [23]. Lipid peroxidation was measured by determining of malondialdehyde (MDA), in plasma samples by high performance liquid chromatography [24]. The results were expressed as μmol/mg protein.

### Assessment of redox status in testis

The testes were homogenized in 0.5 mL of extraction buffer (Tris 50 mM, NaCl 100 mM, EDTA 1 mM, EGTA 2.5 mM, Tween-20 0.1% [pH 7.4], phenylmethylsulfonyl fluoride [PMSF] 100 μg/mL; Sigma-Aldrich) with a Potter homogenizer (Glass-Col K4424; Glas Col, Terre Haute, Indiana) at .050301 *× g*. The samples were then centrifuged at 7820 *× g* for 30 minutes at 4°C.

The Cu-Zn SOD assay is based on the SOD-mediated increase in the rate of autooxidation of catechols in an aqueous alkaline solution in order to yield a chromophore with a maximum absorbance of 525 nm [25]. One Cu-Zn SOD unit is defined as the activity that doubles the auto-oxidation background, and the results are expressed as units/mg of protein. Soluble GSH-Px activity was measured spectrophotometrically in the cytosolic fraction (100000 g supernatant) by the reduction of glutathione disulfide coupled to NADPH oxidation by glutathione reductase [26]. One GSH-Px unit is defined as the activity that oxidizes 1 μmol of NADPH/min and is expressed as units/mg of protein.

The redox status in testicular tissue was assessed by a fluorometric method in order to measure oxidized (GSSG) and reduced glutathione (GSH). The o-phthalaldehyde (OPT) was used as a fluorescent reagent. This method assesses the reaction of GSH with OPT at pH 8 and of GSSG with OPT at pH 12; GSH can be complexed to N-ethylmaleimide to prevent interference of GSH with the measurement of GSSG [27]. The GSH⁄GSSG ratio was then calculated. Lipid peroxidation was assessed by the thiobarbituric acid reaction at pH 3.5, followed by solvent extraction with a mixture of n-butanol/pyridine (15:1, v/v) [28]. Tetramethoxypropane was used as the external standard, and the levels of lipid peroxides were detected spectrophotometrically at 532 nm and expressed as mmol TBARS (Thiobarbituric acid reactive substances)/mg protein.

### SIRTUIN 1 and HIF-1α expression determined by Western Blot (SDS/PAGE)

Aliquots of testis tissue homogenate of testis containing an equal concentration of proteins, 50 μg, were electrophoresed (100 V) on a 12% SDS/PAGE gel as previously described by Farias et al. [10], using a primary mouse SIRTUIN 1 antibody against rat (#8469; 1: 250 dilution) (Cell Signaling, Inc., MA, USA), a primary rabbit HIF-1α (sc-10790; 1:250 dilution) (Santa Cruz Biotechnology, Santa Cruz, CA, USA), a secondary anti-mouse (#7076; 1:1000) (Cell Signaling, Inc., MA, USA) and a secondary anti-rabbit antibody (IC-3R01; 1:1000) (Imuny Rheabiotech, SP, Brazil). Finally, a primary mouse β-tubulin antibody (#3873; 1: 500) (Cell Signaling, Inc., MA, USA) was used as a loading control in all Western blot assays. The bands obtained were analyzed with ImageJ software (http://imagej.nih.gov/ij/) and the integrated density values of the glutathione reductase bands were normalized by dividing by the value of the loading control band.

### Statistical analysis

The Shapiro–Wilk test was used for checking normal distribution of data. The results were presented as mean ± standard deviation (SD). Data were analyzed using the one-way analysis of variance (ANOVA) test followed by Bonferroni analysis. The level of statistical significance was set at p < 0.05 for all tests.

## Results

### PUFA supplementation attenuates pro-oxidant imbalance induced by IHH in plasma

**Figure 1:**
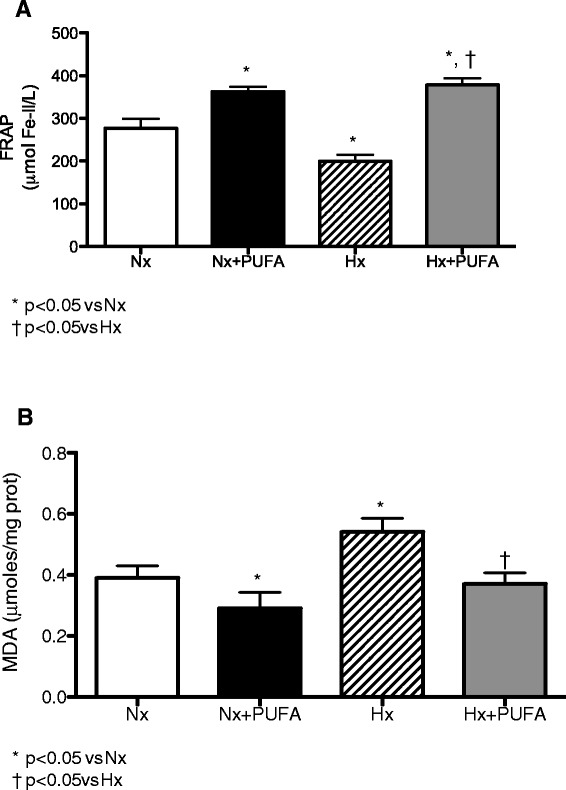
**Effects of PUFA supplementation on antioxidant capacity and lipid peroxidation in plasma.** FRAP levels **(A)** and malondialdehyde (MDA) **(B)** levels were assessed in plasma at the end of study. Normobaric (Nx, n = 7) and intermittent hypobaric hypoxia (Hx, n = 7). Supplemented rats: normobaric, Nx + PUFA (n = 7) and intermittent hypobaric hypoxia, Hx + PUFA (n = 7). Bars indicate mean ± SD. Significant differences: *p < 0.05 vs Nx; ^†^p < 0.05 vs Hx.

After the IHH cycles, FRAP levels show a lower levels than the Nx group (199 ± 39.8 *vs* 276.7 ± 63.4 μmol Fe-II/L) (P < 0.05). PUFA supplementation induces an improvement of antioxidant capacity, showing higher levels of FRAP in Nx + PUFA (362.8 ± 32.1 μmol Fe-II/L) and Hx + PUFA (378.3 ± 44.8 μmol Fe-II/L) (P < 0.05) (Figure 1A). The MDA show a higher levels than the values obtained in the plasma of the Nx group (0.54 ± 0.13 *vs.* 0.39 ± 0.11 μmol MDA/mg prot.) (P < 0.05). PUFA supplementation attenuates these changes in the Nx group (0.29 ± 0.15 MDA/mg prot.) and maintains these values inclusive after IHH exposure (0.37 ± 0.10 MDA/mg prot.) (P < 0.05) (Figure 1B). No significant differences were found between MDA levels in the PUFA group at baseline and following ischemia-reperfusion (Figure 1A-B).

### PUFA supplementation increases antioxidant enzymes activity in testis of rats subjected to IHH

**Figure 2:**
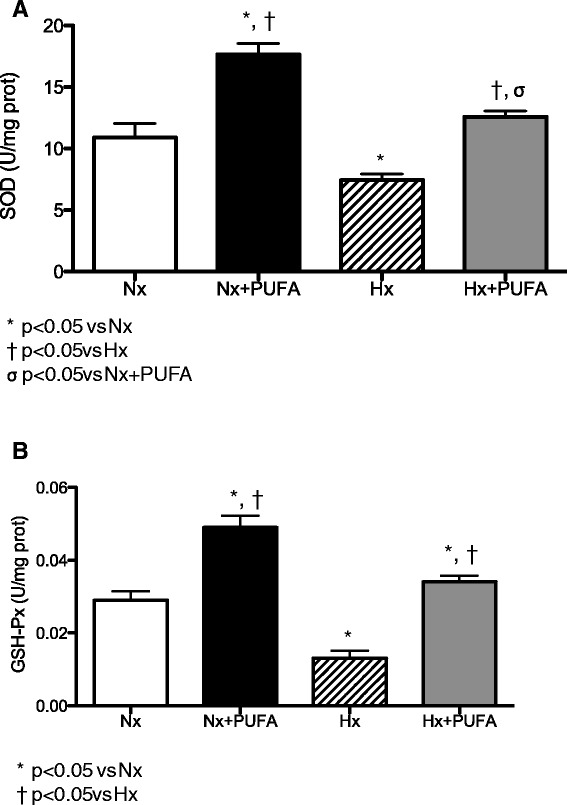
**Effects of PUFA supplementation on antioxidant enzymes activity in testis.** Superoxide dismutase (SOD, **A**) and glutathione peroxidase (GSH-Px, **B**) activities were assessed in testis at the end of study. Normobaric (Nx, n = 7) and intermittent hypobaric hypoxia (Hx, n = 7). Supplemented rats: normobaric, Nx + PUFA (n = 7) and intermittent hypobaric hypoxia, Hx + PUFA (n = 7). Bars indicate mean ± SD. Significant differences: *p < 0.05 vs Nx; ^†^p < 0.05 vs Hx; ^σ^p < 0.05 vs Nx + PUFA.

The SOD and GSH-Px showed lower levels of activity in testis of Hx rats than in the with Nx group (SOD, 10.9 ± 3.23 *vs.* 7.24 ± 1.48 U/mg prot.; GSH-Px, 0.029 ± 0.007 *vs.* 0.013 ± 0.006 U/mg prot.) (P < 0.05). After PUFA supplementation, both the Nx and Hx groups showed higher SOD and GSH-Px activity than the non-supplemented rats (SOD, Nx: 17.66 ± 2.5 U/mg prot. and Hx: 12.57 ± 1.4 U/mg prot.) (GSH-PX, Nx: 0.049 ± 0.009 U/mg prot. and Hx: 0.034 ± 0.005 U/mg prot.) (P < 0.05) (Figure 2A-B).

### PUFA supplementation attenuates pro-oxidant damage in testis of rats subjected to IHH

**Figure 3:**
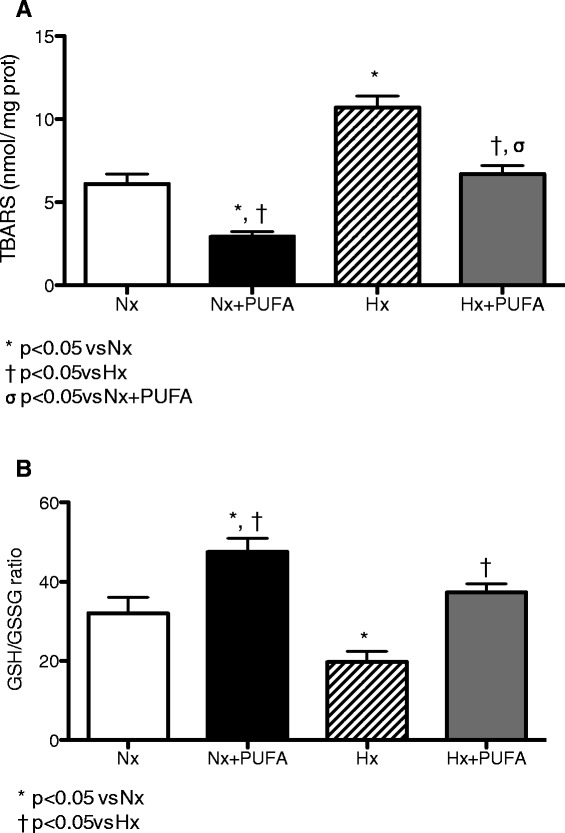
**Effects of PUFA supplementation on oxidative stress markers in testis.** TBARS levels **(A)** and (GSH/GSSG) ratio **(B)** were assessed in testis at the end of study. Normobaric (Nx, n = 7) and intermittent hypobaric hypoxia (Hx, n = 7). Supplemented rats: normobaric, Nx + PUFA (n = 7) and intermittent hypobaric hypoxia, Hx + PUFA (n = 7). Bars indicate mean ± SD. Significant differences: *p < 0.05 vs Nx; ^†^p < 0.05 vs Hx; ^σ^p < 0.05 vs Nx + PUFA.

Lipid peroxidation in testis of Nx + PUFA rats, represented by TBARS levels, was significantly lower compared with levels in the Nx group without supplementation (6.10 ± 1.70 *vs.* 2.90 ± 0.90 nmol TBARS/mg prot.) (P < 0.05). Indeed, IHH induced higher levels of TBARS in testis than those values obtained from testis samples from Hx rats supplemented by PUFA (10.7 ± 1.7 *vs.* 6.63 ± 1.4 nmol TBARS/mg prot.) (P < 0.05) (Figure 3A).

The GSH/GSSG ratio, as a marker of the intracellular antioxidant status, is shown in Figure 3B. The Nx + PUFA group shows higher levels of GSH/GSSG ratio in testis than the Nx group (48.5 ± 9.8 vs 32.3 ± 11.2) (P < 0.05). In the Hx group the values of this ratio were lower than the Nx group (19.7 ± 7.8) (P < 0.05). PUFA supplementation in Hx rats showed higher values of GSH/GSSG than in the Hx rats (37.4 ± 6.2) (P < 0.05). PUFA administration did not affect the SIRTUIN-1 and HIF-1a levels in the Nx group.

### PUFA supplementation induces changes of HIF-1α and SIRTUIN-1 levels in testis after IHH

**Figure 4:**
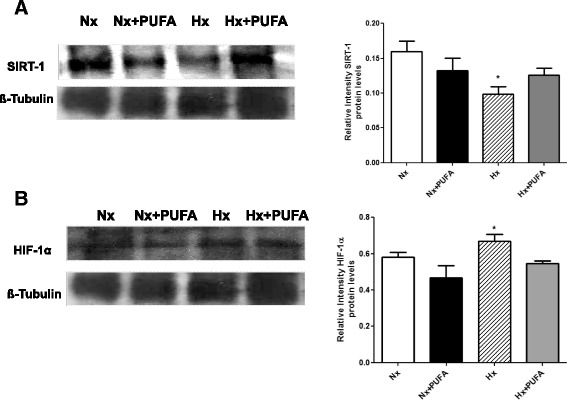
**Effects of PUFA supplementation on SIRTUIN-1 and HIF-1.** Protein Expression Level. **A**: Sirtuin-1 (SIRT-1) and **B**: HIF-1α levels were assessed in testis at the end of study. Normobaric (Nx, n = 7) and intermittent hypobaric hypoxia (Hx, n = 7). Supplemented rats: normobaric, Nx + PUFA (n = 7) and intermittent hypobaric hypoxia, Hx + PUFA (n = 7). Bars indicate mean ± SD. Significant differences: *p < 0.05 vs Nx.

No significant differences in HIF-1α and SIRTUIN 1 testis levels were found between Nx and Nx + PUFA rats. As illustrated in Figure 4, higher levels of HIF-1 and lower levels of SIRTUIN-1 compared to the Nx group were determined (P < 0.05). PUFA administration showed no significant difference in SIRTUIN-1 and HIF-1α levels obtained in the Nx and Nx + PUFA groups, respectively.

## Discussion

Our results demonstrate that the IHH model induced a prooxidant status in the plasma and testis of rats. The oxidative damage was attenuated by chronic supplementation with omega-3 fatty acids. The molecular mechanism of the protective effect of PUFA may involve the induction of antioxidant effects.

The determinations of the time course of oxidative stress markers are important for the study of clinical models associated with IHH, such as pulmonary hypertension and obstructive sleep apnea [29]. Increases in oxidative stress parameters have been demonstrated in a model of shorter human hypoxia exposure, such as *in vivo* lipid peroxidation products (i.e. F2-isoprostanes) and systemic oxidative stress [30,31]. Indeed, antioxidant capacity, assessed through reducing ability of plasma (FRAP) levels has been lower in acute hypoxic exposure compared with normobaric conditions in sports [32,33]. Also, this prooxidant state induced by IHH has been linked to oxidative stress occurrence in local tissues in rats, such as brain, heart, lung and kidney [4,34]. In our study, chronic IHH caused lower FRAP levels (Figure 1A) and higher MDA levels (Figure 1B) in the plasma, these redox changes are consistent with the prooxidant systemic effects induced by our model of IHH. However, the effects of prooxidant imbalance associated with IHH in testis are poorly characterized.

Male Wistar rats have responded to chronic hypoxia with blood and tissue changes, resembling the human response to high altitudes, including polyglobulia and right ventricular hypertrophy [35]. IHH leads to a decrease in the testicular mass, an increase in the interstitial space, and a reduction of the seminal epithelium [9,36]. Recent works have reinforced the existence of an oxidative metabolism in the epididymis of rats subjected to hypobaric hypoxia due to the increased in the regulator enzyme expression of ROS. This rise in ROS production induces an high rate of apoptosis at germinal cell level, leading to a state of hypo-spermatogenesis that may jeopardise masculine fertility [36]. In our study, the vulnerability of male reproductive tissue to oxidative challenges caused by IHH may depend on antioxidant system activity, e.g. the glutathione pathways (glutathione reductase activity) [10]. With regard to these findings, our results are consistent with the tissue prooxidant state, demonstrated by the lower levels of SOD, GSH-Px and GSH/GSSG ratio and the higher levels of TBARS determined in testis of rats subjected to IHH compared with Nx group (Figures 2 and 3).

The hypoxia-induced transcriptional responses are highly regulated in normal development and are deregulated in many diseases [37]. HIF-1 is formed by two subunits: HIF-1α and HIF-1β have been identified as proteins containing a basic helix–buckle–helix and a Per/ARNT/Sim (PAS) domain, which are responsible for the hypoxic induction of erythropoietin. Under reduced oxygen levels, these two proteins heterodimerize in the nucleus and induce the transcription of genes involved in angiogenesis, metabolic and hypoxic adaptation and resistance to oxidative stress and increase invasive properties [38]. In ischemia induced by hypobaric hypoxia, HIF-1α transcriptionally activates hundreds of genes vital for cell homeostasis and angiogenesis. Although potentially beneficial in ischemia, the upregulation of the HIF-1α transcription factor has been linked to inflammation and oxidative stress occurrence [39]. In agreement with this, our results demonstrated higher levels of HIF-1α in testis of rats subjected to IHH compared with the Nx group (Figure 4).

Recent reports have demonstrated that PUFA are beneficial against structural brain and cardiac IR injury [40,41]. Although the protective mechanisms associated with PUFA supplementation are not fully understood, some have suggested that PUFA protection is primarily via anti-inflammatory effects, such as inhibition of monocyte infiltration, NF-kappaB activation and increased biosynthesis of anti-inflammatory active compounds (resolvins-derived from PUFA metabolism) [42]. On another hand, the induction of antioxidant defensive mechanisms is related to direct effects exerted on the cell membrane and induction of genomic responses. This antioxidant activity may act as a homeostatic mechanism counteracting the increased oxidative stress induced by IHH, such as the induction of antioxidant and detoxifying activities [43]. Our results showed that PUFA administration could effectively counteract prooxidant damage, mediated by the attenuation of systemic and tissue oxidative stress induced by IHH. This is reflected by an increase SOD and GSH-Px activities (Figure 2A-B), a reduction in lipid peroxidation and increased FRAP values (Figure 1A). These effects obtained through pharmacological doses (supplementation), confirm the helpful role of PUFA as a promising antioxidant against IHH; however, the type of antioxidant effect, for example scavenger or probable antioxidant compound induction, has not been clearly described.

In the current study, we endeavored to explore some molecular pathways related to the induction of antioxidant responses. SIRTUIN family proteins are involved in specific aspects of metabolism and ROS scavenging proteins, and SIRTUIN-1 and SIRTUIN-3 in particular, have been exhibited effects on antioxidant enzymes activity [44]. Some works show that the upregulation of SIRT1 suppresses the expression levels of pro-inflammatory cytokines and increases the expression levels of SOD and catalase in astrocytes, skeletal muscle and cardiomyocyte, thereby inducing cell protection [45,46]. Experimental evidence suggested that PUFA exert anti-inflammatory functions in both human and animal models by increasing the expression, phosphorylation and activity of the AMPK pathway, which further leads to SIRT1 overexpression [47,48]. However, the antioxidant effects are not well characterized. In our study, no significant differences were found following PUFA administration in the Nx group, and it may be that the dose used (0.3 gkg^−1^d^−1^) was lower than the dose at which exert antioxidant effects in other organs, such as heart, brain and intestinal rat tissues [48,49]. Moreover, the incorporation of these fatty acids into biological membranes was not measured in this study, a significant factor in determining the protective effect against oxidative stress, for example DHA [50,51]. However, the role of other transcriptional pathways cannot be ruled out, for example of Nrf2 dependent signaling pathways. Indeed, our data showed lower levels of SIRTUIN-1 protein levels in IHH rat testis, effects reversed by PUFA supplementation. This would account for a probable mechanism induced by hypoxic insult of short-term not associated with the preconditioning effect [52] (Figure 4).

## Conclusions

In summary, we have concluded that IHH induces a pro-oxidant state in plasma and rat testes through increased lipid peroxidation and reduced in antioxidant mechanisms. PUFA supplementation attenuated these changes by inducing the enzymatic and non-enzymatic antioxidant defense systems. This decreases the possibility of structural and functional damage that need to be characterized. The molecular mechanisms induced by PUFA and IHH in rat testis, therefore should be determined in greater detail.
